# The effect of both a thoracic trauma and a soft-tissue trauma on fracture healing in a rat model

**DOI:** 10.3109/17453674.2011.570677

**Published:** 2011-04-05

**Authors:** Lutz Claes, Anita Ignatius, Raimund Lechner, Florian Gebhard, Michael Kraus, Stefan Baumgärtel, Stefan Recknagel, Gert D Krischak

**Affiliations:** ^1^Institute of Orthopaedic Research and Biomechanics, Center for Musculoskeletal Research; ^2^Department of Traumatology, Hand, Plastic, and Reconstructive Surgery, Center for Surgery, Center for Musculoskeletal Research, University of Ulm, Ulm, Germany

## Abstract

**Background and purpose:**

There is some clinical evidence that fracture healing is impaired in multiply injured patients. Nothing is known, however, about the effects of various types of injuries and their contribution to a possible disturbance of the fracture-healing process. We investigated the effect of a thoracic trauma and an additional soft-tissue trauma on fracture healing in a rat tibia model.

**Methods:**

3 groups of rats were operated: group A with a simple fracture of the tibia and fibula, group B with a fracture and an additional thoracic trauma, and group C with a fracture, thoracic trauma, and an additional soft-tissue trauma. The fracture and the soft-tissue injury were produced by a special guillotine-like device and the thoracic trauma by a blast wave generator.

After one day, the serum level of IL-6 was quantified, and at the end of the study (28 days) the mechanical properties and the callus volume of the healed tibia were determined.

**Results:**

Increasing the severity of the injury caused IL-6 levels to more than double 1 day after injury. It halved the load to failure in mechanical tests and led to reduced callus volume after 28 days of healing.

**Interpretation:**

Fracture healing is impaired when additional thoracic trauma and soft tissue trauma occurs.

Fractures are often associated with other forms of trauma such as additional soft-tissue trauma, blunt chest trauma, or head trauma. Multiply injured patients most often suffer from injuries of the extremities, with two-thirds having fractures and soft-tissue trauma, followed by injuries of the thorax and head (Shorr et al. 1987, Bardenheuer et al. 2000). Multiply injured patients with blunt chest trauma are known to have higher rates of multi-organ failure and mortality (Trupka et al. 1998, Stensballe et al. 2009), while there is some evidence that additional traumas can affect fracture healing. A higher rate of reoperations after fracture fixation was found in multiply injured patients with tibial fractures in comparison to those with isolated fractures (Bhandari et al. 2003). One possible reason might be accumulation of inflammatory factors and the interaction between the inflammatory processes induced by the different injuries.

A blunt chest trauma leads to a rapid systemic inflammatory reaction (Knoferl et al. 2003). Several cytokines (e.g. IL-6, TNF-a, and IL-10) are released systemically within the first few hours after trauma; IL-6 levels appear to correlate best with the severity of thoracic trauma (Knoferl et al. 2003, Liener et al. 2011, Seitz et al. 2011). The same cytokines play an important role during fracture healing, where they regulate inflammation, enchondral bone formation, and remodeling (Cho et al. 2001, Giannoudis et al. 2008). IL-6 was found to be maximally expressed at the early phase of fracture healing whereas IL-1 showed only a moderate change, at a very low level (Cho et al. 2001).

A previous study on fracture healing in rats showed that additional soft tissue damage induced substantially elevated serum levels of IL-6 within 6 hours of injury (Kobbe et al. 2008). Other studies have, however, shown that the effect of a moderate additional soft-tissue trauma only has a temporary (Claes et al. 2006) and small effect on the final fracture-healing outcome (Utvag et al. 2003). To our knowledge, nothing is known about the effect of a blunt thoracic trauma or even a combination of soft-tissue trauma and blunt thoracic trauma on fracture healing.

We tested the hypothesis that these additional traumas would disturb the normal healing process of a fracture. We performed an animal experiment that focused on clinically relevant outcome parameters: bending stiffness, strength of the healed bone, and the amount of callus formation.

## Materials and methods

56 male Wistar rats weighing 250–300 g (Charles River, Sulzfeld, Germany) were used for this study. The experiment was conducted following the regulations for the care and use of laboratory animals and was approved by the German government (Regierungspräsidium Tübingen, no. 814). A simple mid-shaft fracture of the right tibia and fibula was created (group A, n = 16). Another group (B, n = 16) received a blunt thoracic trauma in addition to the fracture, while the third group (C, n = 16) received both the thoracic trauma and a soft-tissue trauma in addition to the fracture. 8 animals each from groups A, B, and C were killed after 1 day for measurement of the early IL-6 reaction. An additional control group (n = 8) was used for comparison of the IL-6 data; these animals received the same anesthesia and pain treatment but no trauma. All the other animals in groups A, B, and C were killed 28 days after trauma to determine the extent of fracture healing.

### Trauma of the extremity

Before application of the traumata, atropine at a dose of 0.05 mg/kg was injected subcutaneously. Anesthesia was subsequently applied intraperitoneally, consisting of 75 mg/kg Ketamine and 12 mg/kg Xylazine.

**Figure 1.**
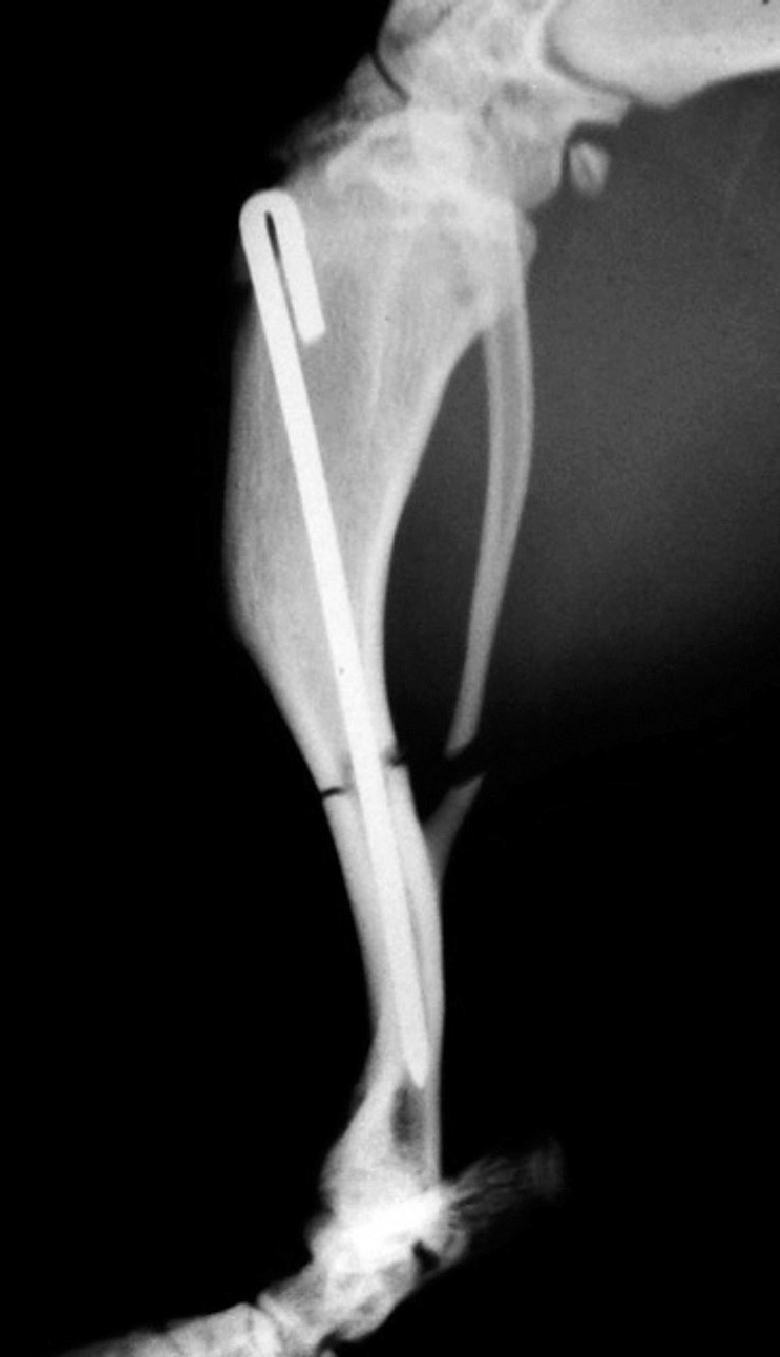
Postoperatively, fracture of the tibia and fibula of the right hind limb of the rat. A Kirschner wire was used to stabilize the tibia.

Before fracture, the medullary cavity of the right tibia was reamed using a Kirschner wire (0.7 mm in diameter) to facilitate the later stabilization of the fracture. After withdrawing the wire again, to the first third of the length of the tibia, a simple closed mid-shaft fracture of the lower right limb without any significant soft-tissue trauma was produced with a 3-point bending guillotine device similar to the apparatus originally described by Bonnarens and Einhorn (1984), with a sharp-edged impact body (weighing 650 g) dropped from a height of 13 cm (impact velocity 1.6 m/s). The site of the fracture had previously been marked with a water-resistant pen 18 mm proximal to the well-palpable malleolus medialis. The distal fragment was re-adapted, the Kirschner wire reinserted, and rotational stability was secured by bending the wire at the proximal end (J-shaped), impacting it carefully into the proximal insertion close to the tuberositas tibiae (Figure 1).

While the rats were still under anesthesia, a high-resolution radiograph of the leg was taken (25 kV, 3 mA, 5 min exposure time; 43805N X-ray; Hewlett Packard, Palo Alto, CA) (film: S400; Kodak, Stuttgart, Germany) (Figure 1).

The additional soft tissue-trauma in group C was performed before fracturing the leg in order to prevent dislocation of the fracture. The soft tissue trauma was set to the dorsal aspect of the lower leg to crush the musculus gastrocnemius at the middle of the lower leg using a modified guillotine apparatus similar to the fracture device, with the leg resting on a flat bearing in the prone position and a blunt impact body. Impact velocity was adjusted to 6 m/s by dropping a weight of 170 g from a height of 180 cm.

### Blunt trauma of the thorax

Blunt thoracic trauma was induced by a single blast wave centered on the thorax with a modified blast wave generator, as previously described (Jaffin et al. 1987, Irwin et al. 1998). The device consists of an upper part that serves as a pressure reservoir, which is separated from the lower nozzle by a 190 μm Mylar polyester film (Du Pont, Bad Homburg, Germany). By opening a high-speed valve, the upper section of the generator is filled with air until the pressure (13 bar) exceeds the resistance of the polyester diaphragm and a reproducible single blast wave is released towards the nozzle. The distance between the generator and thorax was set to 40 mm. All settings for our animal experiment were already established for the rat model by Liener et al. (2011), and generate extensive bleeding in the lung tissue.

### Postoperative care

The animals did not receive any prophylactic antibiotics. They had free access to water and food at all times. Postoperatively, while still under anesthesia, buprenorphine at a dose of 0.05 mg/kg was injected subcutaneously. This was repeated every 12 h on the 3 following days and once a day between days 4 and 6. During the first 12 h after operation, breathing, general condition, and awareness were monitored.

### IL-6 concentration

After 1 day, before killing, a blood sample was taken, cooled on ice, and centrifuged at 2,000 × *g* for 10 min to obtain the serum, which was stored at –80°C. After thawing of the serum, the IL-6 concentration was determined using an ELISA (Rat IL-6 ELISA; R & D Systems Europe, Abingdon, UK) and a photometric system (VERSAmax microplate reader; Molecular Devices, Sunnyvale, CA). 2 samples of each rat serum were measured and the mean value was used for the analyses.

### Microcomputed tomography

After 28 days of healing, the tibias were dissected to remove all soft tissue and the Kirschner wire was withdrawn. Bones were imaged at a resolution of 38 μm using a μCT Fan Beam μ-Scope system (Stratec, Pforzheim, Germany). 3-dimensional reconstructions of the images were visualized using 3-D software (VG Studio Max 1.0; Volume Graphics, Heidelberg, Germany). The bone volume of the whole callus, as well as of the periosteal and endosteal calluses, was determined. The volume of interest was standardized using the outer diameter of the periosteal callus, and in the axial direction using twice the outer diameter of the cortical bone multiplied by a factor of 0.89. This factor was determined from the specimen with the smallest callus in the longitudinal (axial) direction. A global threshold for calcified callus was defined using 25% of the mineral attenuation of cortical bone (Claes et al. 2009).

### Mechanical testing

The flexural stiffness and bending strength of the tibia after 28 days of healing (and removal of the Kirschner wire) was measured using a 3-point bending test. The tibias were potted at their distal and proximal ends in cylinders with poly(methyl methacrylate) to achieve a standardized fixation in the bending device with a 26-mm free length between the bending supports. The bending load was applied in the middle between the 2 supports, at the level of the former fracture line. The bending supports allowed deflection without restrictions by using joints in the bending support. Bending was applied with a materials testing machine (1454; Zwick, Ulm, Germany) at a deflection rate of 1 mm/min until fracture of the bone occurred. The bending load F was recorded continuously against sample deflection (d). The bending test was stopped when a maximum was exceeded in the load/deflection curve for the first time, and this maximum was defined as the failure load. From the linear part of the load/deflection curve, the bending stiffness S was calculated (S = F / d).

### Statistics

The results were analyzed with non-parametric tests (Kruskal-Wallis). If the tests showed significant differences (p < 0.05), differences between groups were analyzed using U-tests. All p-values were adjusted for multiple testing using the Bonferroni procedure. Analyses were performed using standard statistical software (JMP; SAS Institute Inc., Cary, NC).

## Results

**Figure 2.**
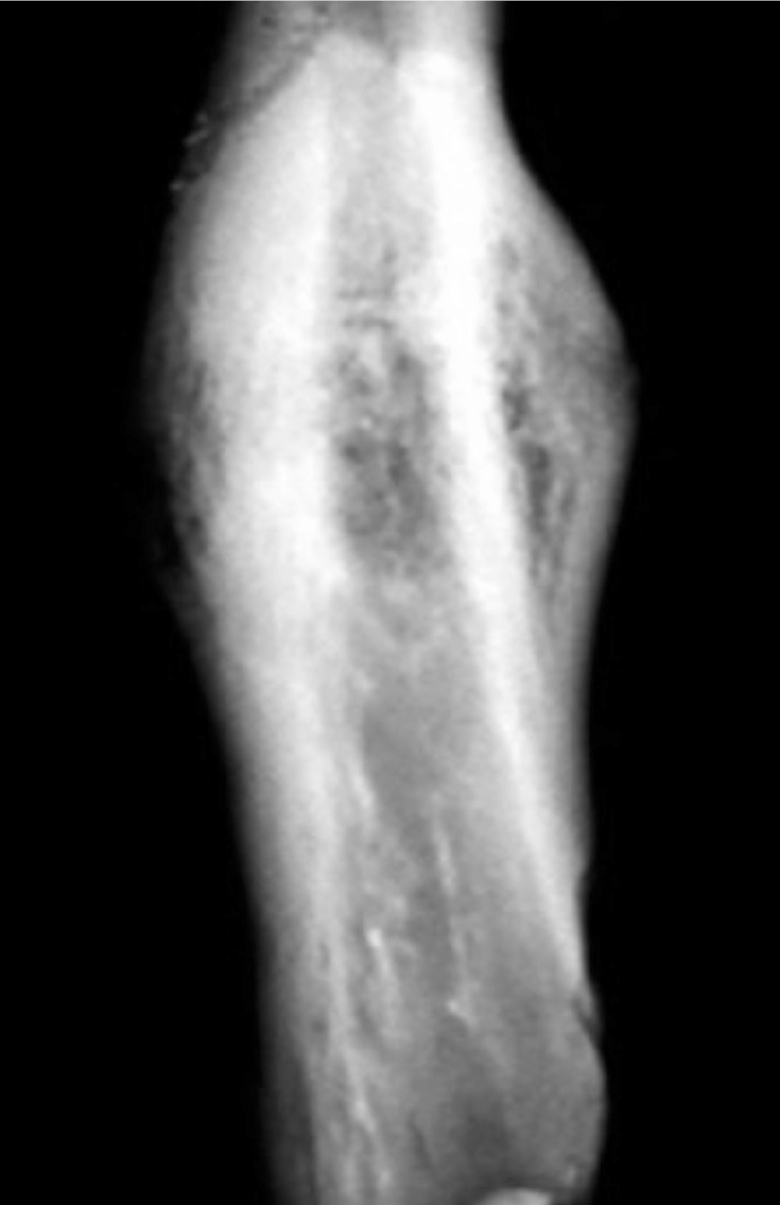
Bone healing 28 days after creation of a simple fracture and internal fixation; after removal of the Kirschner wire. Callus formation has bridged the fracture line.

Due to complications during anesthesia and thorax trauma, and because of the occurrence of complex fractures (not simple transverse fractures, but involving more than 2 fragments) 6 rats had to be excluded from the study. For the fracture healing study, 6 rats in group A, 7 rats in group B, and 6 rats in group C remained. Thirty-one rats could be used for the analysis of the IL-6 concentration at day 1 after trauma. One animal in the control group died during anesthesia, leaving 7 animals in the control group. Most of the rats tolerated both the trauma and the operation well, and regained full activity between the second and third day after trauma. The bone healing showed the characteristic image of a callus healing under flexible fixation (Figure 2).

The soft-tissue trauma led to swelling of the lower leg, which had its maximum after 1 day and disappeared after 7 days.

### IL-6 concentration

Serum IL-6 concentration was statistically significantly increased in groups B and C one day after trauma (Table) relative to the control rats without trauma, which had a mean IL-6 concentration of 15 (SD 3.4) pg/mL.

**Table:** Results of the μ-CT, biomechanical test, and IL-6 determination. Mean values (SD)

	Group A	Group B	Group C
Callus volume, total (mm^3^)	87 (16)	74 (25)	61 (13)
Callus volume, periosteal (mm^3^)	82 (15)	70 (24)	58 (13)
Failure load (N)	41 (12)	30 (7)	21 (9) **^a^**
Bending stiffness (N/mm)	45 (18)	38 (23)	30 (15)
IL-6 (pg/mL)	27 (5)	34 (18) **^b^**	48 (28) **^b^**

Group A: fracture; group B: fracture and thoracic trauma; group C: fracture, thoracic trauma, and soft-tissue trauma.

**^a^** p< 0.05 in comparison to group A.

**^b^** p< 0.05 in comparison to control group.

### Microcomputed tomography

With increasing severity of the injury, the amount of total callus volume and periosteal callus volume decreased (Table). There was more periosteal callus formation in group A than in group C, which showed the lowest amount of periosteal callus. The total callus formation showed similar results. In contrast, the endosteal callus volume was similar between the groups (results not shown). The reason for this was that the endosteal cavity was mainly filled by the Kirschner wire in all groups.

### Mechanics

The failure load decreased with increasing severity of the injury (Table). In comparison to group A, the failure loads of group B and of group C were statistically significantly reduced (Table). The bending stiffness showed the same trend, but the groups did not show any statistically significant differences (Table).

## Discussion

We found that fracture healing was increasingly impaired with increasing severity of trauma. In particular, the application of a soft tissue trauma in addition to thoracic trauma had a dramatic effect. One reason for this effect was the reduced callus formation in these rats in comparison to animals with isolated fractures. This effect was even more pronounced when a soft-tissue trauma was also applied. In particular, the periosteal callus volume, which contributes most to the mechanical stability of the healing bones, was substantially reduced in the groups with additional thoracic and soft-tissue trauma.

We have found similar effects in a previous study in which we compared fracture healing with and without a soft-tissue injury (Claes et al. 2006). Immediately postoperatively, the blood supply was more suppressed with an additional soft-tissue trauma and the amount of callus formation after 7 days was reduced by about one third in the group with additional soft-tissue trauma. After 28 days of healing, however, the early disturbance of the healing process by the soft-tissue trauma was no longer evident.

In this study, the soft-tissue injury we applied in addition to the fracture and the thoracic trauma had a statistically significant effect on the fracture healing process. One possible explanation might be that the effects of soft-tissue traumas are cumulative with respect to injury burden. The effect of one additional trauma such as the soft tissue injury in the previous study (Claes et al. 2006) or the thoracic trauma in the present study did not have statistically significant effects on the final outcome at day 28, however. The application of a thoracic trauma and a soft-tissue trauma together did, however, lead to a statistically significant difference in healing outcome at day 28 (Table). In comparison to an isolated fracture, the combined trauma caused a reduction in load to failure of the healed bones, and a smaller periosteal callus volume. These results are in accordance with those of clinical studies, which have shown a higher rate of reoperations in patients with tibial fractures when they have multiple injuries (Bhandari et al. 2003).

The first days of fracture healing in rats represent an inflammatory stage (Einhorn 2005). During this stage especially, intercellular communication has been shown to involve proinflammatory cytokines such as IL-1, TNF-a, and IL-6. They play a crucial role in initiation of the repair process (Einhorn et al. 1995). Cytokines also regulate endochondral bone formation, the predominant form of callus healing during intramedullary fixation of fractures, as in this experiment (Barnes et al. 1999).

1 day after trauma, the systemic IL-6 levels in the fracture group were approximately twice as high as in the control group (without injury), whereas they were three times higher and therefore statistically significantly greater than control values when thoracic trauma and soft-tissue trauma were both applied. This is in accordance with clinical data from multiply injured patients, which have shown an increased level of IL-6 within the first 24 h (Giannoudis et al. 2008). The elevated levels of IL-6 could be at least partly responsible for the observed impairment of fracture healing. IL-6 has multiple concentration-dependent effects on bone cells. IL-6 transgenic mice showing chronic overexpression of IL-6 developed a severe osteopenia with reduced osteoblast and increased osteoclast numbers and activity (De Benedetti et al. 2006). Callus mineralization and maturation are delayed during fracture healing in IL-6 (–/–) mice, indicating an essential role of IL-6 during fracture healing (Yang et al. 2007). IL-6 may also be a potent stimulator of bone resorption, by its ability to increase osteoclast formation from precursor cells (Jilka et al. 1992). One possible reason for the larger effect of the combined trauma to the healing outcome might be a larger effect on IL-6. The fast and transient posttraumatic systemic increase in IL-6 could therefore influence the early fracture healing process locally and contribute to delayed healing.

A limitation of this study, however, is that we determined only IL-6 levels to follow the early inflammatory process. In the complex cascade of events after trauma, a large number of cytokines are involved (Cho et al. 2001, Knoferl et al. 2003), but IL-6 concentration appears to play the most important role and correlates best with the severity of thoracic trauma (Gebhard et al. 2000, Seitz et al. 2011). However, other cytokines—e.g. TNF-a or IL-10—might also influence fracture healing locally. As a first step, we concentrated on IL-6 to characterize changes in the level of inflammation due to trauma and to find any correlations with clinically relevant fracture healing outcome parameters, such as strength and callus volume of the healed bone. The molecular and cellular mechanisms that were responsible for the observed impairments in bone regeneration remain to be determined.

The mechanical outcome was measured as failure load and calculated as bending stiffness. In this experiment, the load to failure was the better factor because it showed lower standard deviations in the data. The explanation for this effect is that in a later phase of fracture healing, the determination of bending stiffness is more sensitive to imprecision (Chehade et al. 1997). Despite these limitations, our study shows that in this model system fracture healing was impaired by an additional thoracic trauma and that this effect was even more pronounced when also accompanied by a soft-tissue trauma.
